# Neural underpinnings of open-label placebo effects in emotional distress

**DOI:** 10.1038/s41386-022-01501-3

**Published:** 2022-12-01

**Authors:** Michael Schaefer, Anja Kühnel, Felix Schweitzer, Sören Enge, Matti Gärtner

**Affiliations:** Medical School Berlin, Berlin, Germany

**Keywords:** Psychology, Emotion

## Abstract

While placebo effects are well-known, research in the last decade revealed intriguing effects that placebos may have beneficial effects even when given without deception. At first glance, this seems paradoxical, but several studies have reported improvements in pain, depression, or anxiety. However, it still remains unclear whether these results represent objective biological effects or simply a bias in response and what neural underpinnings are associated with the open-label placebo effects. In two studies, we address this gap by demonstrating that open-label placebos reduce self-reported emotional distress when viewing highly arousing negative pictures. This reduced emotional distress was associated with an activation of brain areas known to modulate affective states such as the periaqueductal gray, the bilateral anterior hippocampi, and the anterior cingulate cortex. We did not find any prefrontal brain activation. Furthermore, brain activation was not associated with expectation of effects. In contrast, we found that brain responses were linked to general belief in placebos. The results demonstrate that the neural mechanisms of open-label placebo effects are partly identical to the neurobiological underpinnings of conventional placebos, but our study also highlights important differences with respect to a missing engagement of prefrontal brain regions, suggesting that expectation of effects may play a less prominent role in open-label placebos.

## Introduction

At least since Henry K. Beecher’s famous article about the “powerful placebo” it has been known that placebo pills or interventions can have beneficial effects [1]. While the idea of placebos is certainly much older, Beecher’s publication marks perhaps the first major scientific acknowledgment of placebos as a potential treatment [2]. Since then, it is widely accepted that a placebo treatment can have a significant impact for a wide variety of symptoms [3]. Unfortunately, placebos have a major disadvantage. Since deception is thought to be crucial, treatment with placebos is associated with severe ethical problems, for example, the undermining of informed consent, respect for persons, and trust between patient and healthcare provider. Until very recently, the idea of giving placebos without deception would have been considered ridiculous. However, there is an increasing body of evidence that even prescribing placebos when patients know they are receiving placebos (open-label placebos, OLP) may help patients with clinical disorders and individuals with nonclinical symptoms. OLPs have been shown to have beneficial effects in a variety of symptoms based on patient subjective reports, including, for example, irritable bowel syndrome, depression, pain, anxiety, and emotional distress [4–9].

However, although several studies showed positive, counter-intuitive findings, it still remains unclear whether OLP responses describe objective psychobiological effects or perhaps simply represent response bias. In contrast to conventional placebos it is obvious that OLP paradigms cannot be double-blinded, therefore it is difficult to rule out response bias when using only self-report measures. To date, very little work has attempted to measure the effects of OLPs using objective physiological outcomes, with mixed results. For example, Mathur et al. examined whether OLPs affect wound healing and found no results [10]. Leibowitz et al. investigated physiological allergic responses and found no direct main effects, but an interaction with the belief in placebos ([11] similar [12]). Guevarra et al. employed an electroencephalographic (EEG) approach to examine emotional distress in healthy subjects and reported OLP effects on EEG markers for both, neutral and negative stimuli [13].

Moreover, it remains unclear whether the possible psychological mechanisms due to OLP treatments are similar to conventional placebos, which are explained with classical conditioning [14, 15], patient’s expectations [16], or social interaction with healthcare practitioners [14]. Which (if any) of these mechanisms explain the effect of OLPs remains to be cleared. This also applies to the neural mechanism underlying OLP responses. Neuroimaging studies have provided important contributions to our understanding of the way how placebos with deception work. For example, placebo analgesia has been shown to engage the descending pain modulatory network, including the dorsolateral prefrontal cortex (DLPFC), the anterior cingulate cortex (ACC), insula, somatomotor brain regions, amygdala, and the periaqueductal gray (PAG) [17–19]. This endogenous pain modulation circuitry includes opioid responses in the brainstem and amplifies or inhibits incoming pain signals [18]. Similar networks are discussed for emotional placebo responses [15, 18]. Again, it remains unclear whether OLP responses recruit similar brain regions.

The present study addresses these questions by examining neural correlates of OLP effects. We decided to investigate OLP effects on emotional distress, since this context has been shown to respond to both deceptive and non-deceptive placebo treatment [7, 13, 20–23]. The aim of the study was to test whether an OLP treatment affects not only self-report measures but also objective psychophysiological processes related to stress perception and modulation, and to identify the underlying neural mechanisms of OLP responses. Therefore, we examined OLPs by employing an fMRI approach. Some previous studies addressed these questions by using different biological markers and approaches including, for example, EEG, with mixed results ([10, 11, 13]. However, the present study is the first to employ fMRI to unravel the neural substrates of OLP responses, an approach that promises high spatial resolution.

In a first study, we studied the behavioral effects of an OLP treatment on self-reported emotional distress when viewing negative emotional pictures. Experiment 2 replicates this paradigm within an fMRI approach to understand the neural underpinnings of these OLP effects. In both experiments participants were randomly assigned to one of two groups and subsequently given a nasal spray with saline solution. In the OLP group the participants were told that this spray was a placebo with no active ingredients but would help to reduce their negative feelings when watching the distressing pictures. Participants in the control group received the same spray but were told that the spray was necessary due to technical requirements for performing the experiment (see Fig. S1 in Supplementary Material). The paradigm was adopted from [13]. Our first study was identical to [13], while our second experiment used an fMRI approach, in contrast to [13] which employed EEG.

For study 1, we hypothesized that participants in the OLP group reported less emotional stress when viewing the pictures. For study 2, we assumed that the reduced emotional stress is reflected by engaging a network of brain regions previously associated with placebo effects [17–19], in particular with respect to the reduction of emotional distress induced by unpleasant pictures (e.g., orbitofrontal cortex, ACC, and PAG) [21].

## Materials and methods

### Participants

In study 1, our behavioral experiment, 112 healthy individuals participated (mean age 23.53, ±7.32 years, 67 females). Study 2 was an fMRI experiment and included a new sample of 44 participants (22.34 ± 2.62 years). This sample included only female participants to control for sex differences in emotion processing and regulation ability [24, 25].

All participants gave written informed consent and had no neurological or psychiatric history (self-reported). The study adhered to the Declaration of Helsinki and was approved by the local human subjects’ committee.

The study was introduced to the participants as an experiment on psychophysiological interactions.

### Procedure study 1

In study 1 participants were randomly assigned into two groups. Participants in the OLP group first read a presentation about placebo effects, how powerful they can be, and that even placebos without deception have been shown to have effects (based on [13]). At the end of the presentation, the experimenter told the participants that they will be given “a placebo nasal spray to reduce your negative emotional reactions. Again, this is a placebo, which means it does not contain any active ingredients, only saline solution, and it is completely harmless. But as you have read from the presentation, if you believe that the nasal spray will reduce your negative emotional reactions, then it actually will.“ (taken from [13]). Then the experimenter gave the participant a saline nasal spray (one application to each nostril). The spray was labeled as a “Placebo” and depicted the logo of the university.

The control group read a presentation about neurological processes of pain and the treatment of this pain. Both presentations were matched with respect of valanced words, length, and other features. The material was adopted from Guevarra et al. [13]. At the end of the presentation participants in the control group then received the saline nasal spray, but here we explained this spray as necessary to help obtain better physiological readings [13]. The nasal spray was labeled as “Nasal spray” with the logo of the university. Participants of the control group were told that this spray contained only saline solution. Furthermore, participants in the control group were not aware that participants in the other group received a “Placebo”-Spray (or that the study was about placebo effects), thus, they could not be disappointed to be placed in the control condition.

After the application of the nasal spray, participants began the picture viewing task. The task was based on previous studies on processing of emotional distress [13, 21]. Participants viewed 30 negative and 10 neutral pictures in a randomized order. The pictures were balanced with respect to normative valence and arousal ratings and taken from the IAPS data base [26] (see Supplementary Material; images were identical to Guevarra et al.). Each picture was presented for 6000 ms, followed by a fixation cross (4000 ms) and a picture rating period for 5000 ms. During this rating period participants were asked how the image made them feel on a nine-point Likert-scale ranging from 1 (not at all negative) to 9 (very negative).

After the picture viewing task, participants were asked to answer additional questions related to the expectation of the nasal spray (using a VAS scale, see Supplementary Material) and the quality of the presentation (to test for differences with respect to the presentation; six items asking to what extent the information in the presentation was effectively conveyed, convincing, novel, interesting, well-written, and useful; analogue to [13]).

Statistical analyses were performed using a mixed-factorial ANOVA with group as a between-subjects (OLP, control), and picture type as within-subjects factor.

### Procedure study 2

Study 2 replicated study 1 within an fMRI context and using an independent sample. Analogue to study 1 we first randomly assigned the participants to two groups, an OLP and a control group. The subsequent preparation process was identical to study 1 (reading the presentation and providing the nasal spray, see also Fig. S1). The detailed instruction while giving the nasal spray to the participants can be found in the Supplementary Material (S10).

After receiving the nasal spray participants entered the scanner. In the fMRI the participants viewed 45 negative and 45 neutral pictures in a randomized way (identical to Guevarra et al. [13] plus additional pictures taken from IAPS data base [26], see Supplementary Material). Each image was presented for 4 s and followed by a fixation cross of 12 s. Pictures were shown in three blocks. In the break between the blocks participants received again the nasal spray (once in each nostril), resulting in a total of six nasal spray applications for each participant. At the end of the experiment, we asked the participants to rate how negative the pictures made them feel by using a key with four buttons (Likert-scale ranging from 1 to 4, 1 = not at all negative, 4 = very negative). Analogue to Guevarra et al. participants did not report their feelings immediately after each picture to obtain pure neural responses without any possible introspective processes [13, 27].

The experiment lasted for about 45 min. Images were presented on a screen at the end of the scanner bed. Participants viewed these stimuli through a mirror mounted on the birdcage of the receiving coil.

After scanning we asked the participants to complete questionnaires with respect to the expectations (identical to study 1) and the belief in OLPs (5 questions embedded in more general questions, see Supplementary Material, identical to [13]), the general belief in placebos [11] (see Supplementary Material), dispositional optimism (LOT-R) [28], trait anxiety (STAI [29]), and social desirability ratings (SES [30]). Finally, participants had to assess the quality of the presentation (see study 1) and were examined with respect to the perceived warmth or competence of the experimenter (to test for differences with respect to the group; questions on competence of the experimenter, knowledge of what he was doing, authority, understandability, self-confidence, sympathy, warmth, coldness; analogue to [13]).

FMRI data were acquired using a 3 T Siemens Tim Trio scanner (Siemens, Germany). Six participants were scanned with an updated system to a Magnetom 3 T Prisma Fit (those subjects were divided equally between the OLP and control conditions). High-resolution T1-weighted structural images for anatomic reference were acquired using an MP-RAGE sequence (TR = 1650 ms, TE = 5 ms). Functional images were collected using gradient echo-planar images (TR = 2 sec, TE = 35 ms, flip angle = 80 degrees, FOV = 224 mm, number of slices = 32, voxel size = 3.125 ×3.125 mm).

Preprocessing of imaging data was done using the Statistical Parametric Mapping Software (SPM12, Wellcome Department of Imaging Neuroscience, University College London, London, UK). For each subject these steps included realignment to correct for inter-scan movement, sinc interpolation, normalization into a standard anatomical space (MNI, Montreal Neurological Institute template, resulting in isotropic 3 mm voxels), and finally smoothing with an 8 mm FWHM Gaussian kernel (full-width half maximum) (standard preprocessing pipeline, [31]).

Statistical parametric maps were then computed using multiple regressions with the hemodynamic response function modeled in SPM. We first examined data on the individual subject level by calculating brain responses while participants viewed negative relative to neutral images (fixed-effects-model). Then we used the resulting parameter estimates for each regressor at each voxel for second-level analysis (random effects model), in which we compared those contrasts with respect to both conditions (OLP relative to control).

Results of whole-brain analyses of brain activation (OLP relative to the control group and vice versa) were reported when surpassing the voxelwise-threshold of *p* < 0.001 (uncorrected). In addition, we analyzed brain activation with small volume correction (SVC) for a priori regions of interest (ROIs) (at *p* < 0.05, FWE corrected within these ROIs). These ROIs were based on brain regions reported in previous research on placebo effects and emotion processing and included bilateral amygdala [21], PAG (spheres of 5 mm), the right orbitofrontal cortex, right insula, ACC, bilateral DLPFC [32], as well as the hippocampi [33] (spheres of 10 mm). For the general contrast negative relative to neutral pictures (irrespective of groups), we report activations with voxelwise-threshold of *p* < 0.001 (uncorrected) as well as ROIs of amygdala activations based on previous research [21] (small volume correction, sphere of 5 mm, at *p* < 0.05, FWE corrected within these ROIs). Anatomical interpretation of the functional imaging results was performed by using the SPM anatomy toolbox.

## Results

### Study 1: OLP and self-reported emotional distress

**Fig. 1:**
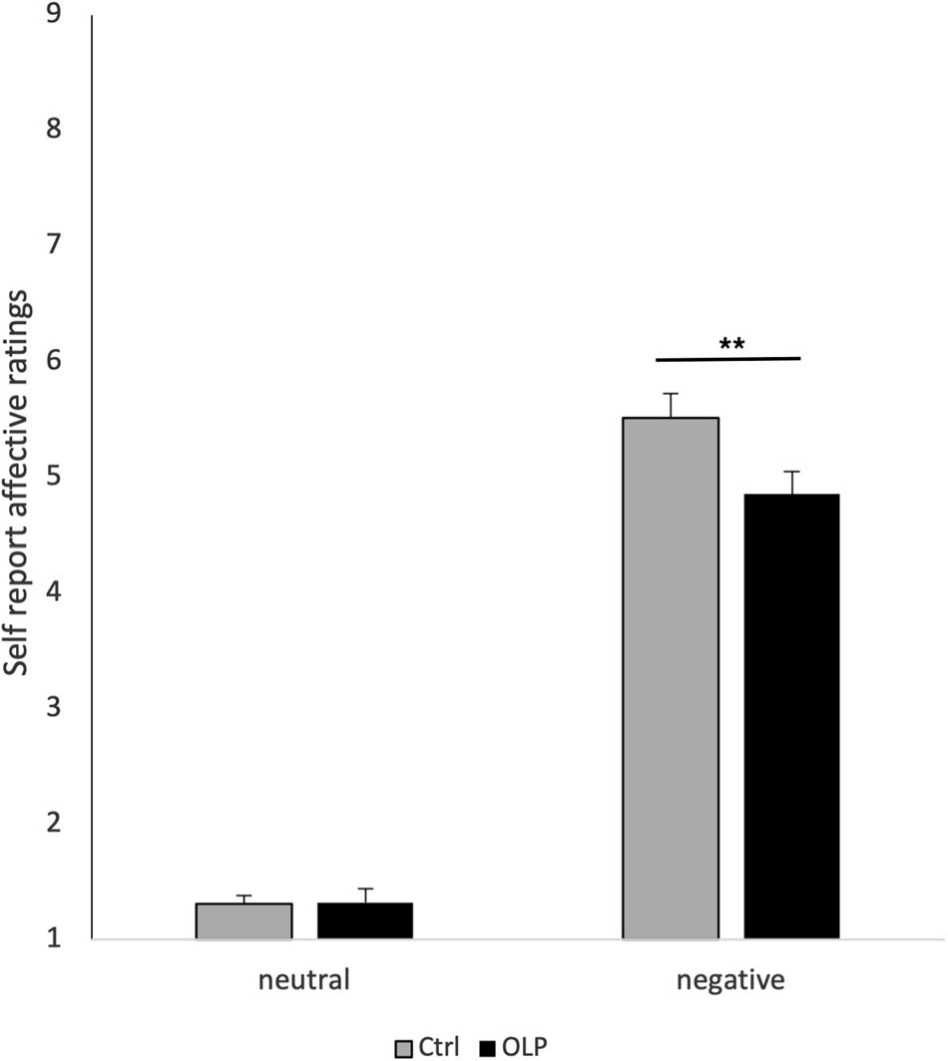
Results of study 1. See text for details.

When analyzing self-reported emotional distress, a mixed-analysis ANOVA revealed a main effect of picture type (F (1,110) = 742,41, *p* < 0.001, partial eta^2^ = 0.87), indicating that the negative images were indeed rated as negative. Furthermore, we found a significant interaction with group (F (1,110) = 5.54, *p* = 0.020, partial eta^2^ = 0.05). A subsequent post-hoc test demonstrated that participants in the OLP group experienced fewer negative feelings than the control group (control: 5.51 ± 1.56, OLP: 4.85 ± 1.49: t (110) = 2.30, *p* = 0.011, Cohen’s d = 0.43; one-sided). For neutral pictures we did not find any group differences (t (110) = −0.03, *p* > 0.10) (see Fig. 1).

When asking the participants whether they think that the nasal spray had reduced their emotional distress (expectation), participants in the OLP group agreed to this statement stronger than the control group, suggesting that the manipulation of the information with respect of the effectiveness of the placebo nasal spray had worked (t(110) = −2.05, *p* = 0.021). The degree of the strength of this expectation was not linked to the reported emotional distress (*p* > 0.10).

### Study 2: Neural correlates for OLP effects

While study 1 demonstrated that the placebo nasal spray successfully reduced emotional distress when viewing negative images, study 2 aimed to determine the neural underpinnings of this effect.

OLP and control group did not differ with respect to demographic data or other variables (optimism, anxiety, social desirability ratings, or general belief in placebos; see Table 1). In addition, there were no differences between the groups with respect to the evaluation of the quality of the presentation (which the groups received to manipulate the effectiveness of the nasal spray before the experiment) or the perceived warmth or competence of the experimenter. A manipulation check revealed that the OLP group believed more in the power of OLPs than the control group, as intended by our experimental setup (t(42) = 2.41, *p* = 0.011). When asking the participants after the experiment whether they thought that the nasal spray may have reduced their emotional responses towards the negative pictures (expectation), we found no results with respect to the groups (t(42) = 0.10, *p* > 0.10). This is in contrast to the first study and may be explained by the relatively long time (several minutes) between the ending of the experiment and the time participants completed the questionnaire (outside the scanner).

**Table 1:** Behavioral results for study 2.

	CTRL group	OLP group	
***N***	21	23	
age	22.20 ± 2.04	22.45 ± 3.05	
evaluation of presentation quality	7.27 ± 1.25	7.20 ± 1.57	t(42) = −0.16, *p* > 0.10
evaluation competence of experimenter	6.30 ± 0.56	6.22 ± 0.62	t(42) = −0.44, *p* > 0.10
evaluation warmth of experimenter	6.07 ± 0.75	5.88 ± 0.78	t(42) = −0.78, *p* > 0.10
belief in OLPs	**5.35** ± **3.11**	**7.49** ± **2.77**	**t(42)** = **2.41**, ***p*** = **0.011**
expectation (effectiveness of nasal spray)	2.42 ± 2.44	2.35 ± 1.96	t(42) = 0.10, *p* > 0.10
general belief in placebos	7.41 ± 1.65	7.32 ± 2.23	t(42) = −0.15, *p* > 0.10
optimism	9.05 ± 2.82	8.48 ± 2.73	t(41) = −0.68, *p* > 0.10
trait anxiety (STAI)	47.24 ± 5.40	47.39 ± 4.62	t(42) = 0.10, *p* > 0.10
social desirability responding (SES)	23.90 ± 2.32	24.78 ± 1.91	t(41) = 1.37, *p* > 0.10

Bold data point to significant differences. See text for further details.

Behavioral results replicated the results of the first study by demonstrating that OLPs reduced self-report emotional distress (controls: 2.04 ± 0.62, OLP: 1.60 ± 0.76 on a scale ranging from 1 to 4 with 4 as feeling extreme distress; t (42) = 2.09, *p* = 0.021, Cohen’s d = 0.63). Self-reported emotional stress in the OLP group was not linked to the expectation of the participants (*r* = −0.16) or the belief in OLPs (*r* = 0.20). Furthermore, it was not linked to the general belief in placebos or optimism (all *p* > 0.10, see Table S4, Supplementary Material). The control group also lacked a correlation between emotional distress and expectancy (*p* > 0.10), but the question may have appeared odd for the participants, given that the nasal spray was introduced to them as technically necessary.

**Fig. 2:**
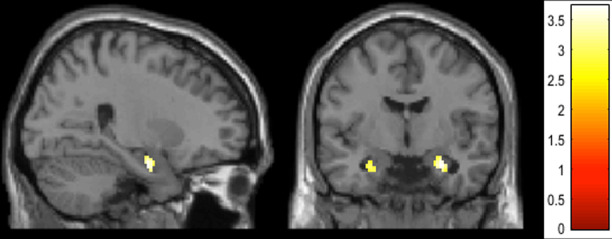
Results of study 2. Statistical maps showing brain activation of amygdala when participants watched negative relative to neutral pictures (irrespective of groups) (based on ROI analysis, picture shows activation at *p* < 0.005 uncorrected, for picture purpose only).

Comparing brain responses to negative images (compared with neutral pictures) irrespective of groups revealed activation in right amygdala (based on ROI analysis, FWE corrected, at *p* < 0.05) as well as the superior temporal gyrus and other areas (based on whole-brain analysis, *p* < 0.001, uncorrected), as expected (see Fig. 2 and Supplementary Table S3).

**Fig. 3:**
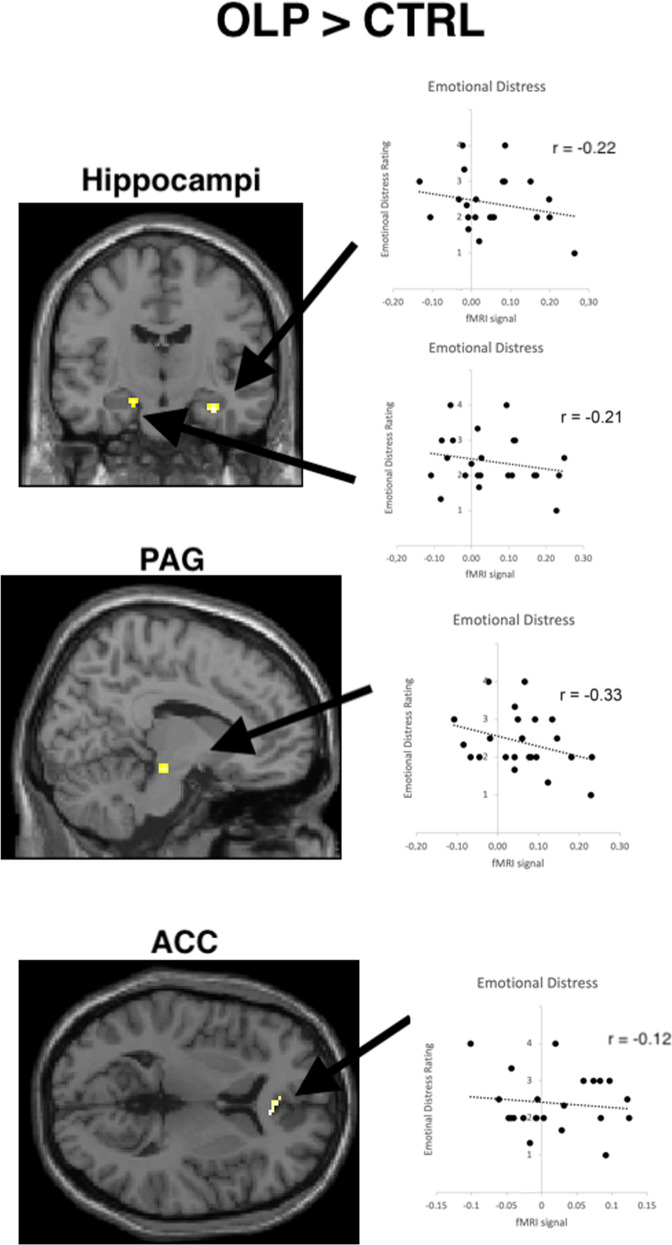
Results of study 2. Brain activation associated with open-label placebos relative to control revealed engagement of hippocampi and periaqueductal gray (based on ROI analyses, FWE corrected at *p* < 0.05, see also Table 2 and text). No brain regions in prefrontal cortex were found even with a lenient threshold of *p* < 0.01 (uncorrected). Areas of significant fMRI signal change are shown as color overlays on the T1-MNI reference brain (picture shows activation at *p* < 0.005 uncorrected, for picture purpose only).

When comparing brain responses for OLP relative to control group we found activated clusters in PAG and bilateral hippocampi (ROI-based analysis, FWE corrected, at *p* < 0.05; exploratory whole-brain analyses at *p* < 0.001 (uncorrected) revealed the same brain regions but no additional activation) (see Table 2 and Fig. 3). These brain activations were negatively linked to the felt distress in the OLP group (see Table S4 in Supplementary Material). When lowering the threshold to *p* < 0.005 (uncorrected, whole-brain analysis) results demonstrated additional activation in the ACC, but not in other brain regions. In particular, we did not find brain activity in prefrontal brain regions (even with a very lenient threshold of *p* < 0.01, whole-brain analysis, uncorrected).

**Table 2:** Results of random effects analysis for brain responses when participants viewed negative relative to neutral pictures (ROI analyses at *p* < 0.05, FWE corrected, and additional brain activations for exploratory whole-brain analyses at *p* < 0.005, uncorrected, in brackets).

	Brain region	Peak MNI location (x, y, z)	Peak z-value
OLP > Control	R Hippocampus	32 −14 −22	3.64
	PAG	12 −22 −16	3.36
	(ACC)	12 28 6	3.28
	L Hippocampus	−18 −16 −18	3.08
Control > OLP	(L supramarginal gyrus)	−54 −42 48	2.95

The contrast control relative to OLP group showed no active clusters in ROIs or whole-brain analysis (at *p* < 0.001, uncorrected). When lowering the threshold to *p* < 0.005 (uncorrected, whole-brain analysis) we found brain activation in left supramarginal gyrus, but no other clusters (see Table 2).

Placebo-related brain activations in hippocampi and PAG were not related to the expectation that OLPs would work (expectation) or to the belief in OLPs (all *p* > 0.10), but with the general belief that placebos do work (right hippocampus: *r* = 0.37, *p* = 0.085, left hippocampus: *r* = 0.40, *p* = 0.056, PAG: *r* = 0.61, *p* = 0.002) (see Supplementary Materials, S4, S5). There were no relationships between placebo-related brain activation and personality variables (optimism, anxiety, social desirability ratings; all *p* > 0.10).

## Discussion

Numerous studies in patients and in healthy subjects reported effects of OLP treatments [8, 9, 34]). Most of those studies used self-report measures to determine effects. Given that OLP paradigms cannot be blinded, it remains unclear whether the effects may be explained by response bias (e.g., social desirability) or represent genuine changes in objective psychophysiological processes. Employing neuroimaging approaches may help to address this issue.

We report that an OLP treatment reduced self-reported measures of emotional distress in healthy subjects, which is in line with previous literature on effects of OLPs on emotional distress or anxiety (e.g., [7, 12, 13]). Moreover, we also replicated this beneficial effect of OLPs on emotional distress in an fMRI environment, allowing us to understand the neural mechanisms underlying the OLP effect. We found the effect to be predominantly associated with an activation of the hippocampi, the ACC, and the PAG. A role of the PAG in the network of brain areas representing a placebo response is well-known, for example, in the descending pain modulatory network in placebo analgesia. The PAG includes many opiate neurons with descending spinal efferents, which are thought to modulate the pain perception in placebo responses even at a spinal level [32, 35]. Similar mechanisms are also discussed for emotional placebo responses [15]. Here we argue that the PAG may have modulated emotional distress in the amygdala. It is known that there is a pathway from amygdala to the PAG, which in animals directs appropriate fear-related behavior such as freezing or fleeing. Remarkably, this pathway has been shown for innate and learned fear responses [36]. Together with an engagement of the ACC, which previously has been linked to placebo responses (for example, for emotional distress when viewing unpleasant pictures [21]), the PAG is a well-known key structure in the network of brain regions underlying the placebo reaction.

We also found the hippocampus to be linked to the OLP effect. This region is not a typical part of the network of brain structures representing a placebo response (but interestingly in nocebo effects [37–39]). The hippocampus is known to play crucial roles in regulating affective conditions and thereby representing a central hub for the emotional brain [40]. It is part of a network including PAG and amygdala which represents anxiety responding [39, 41]. For example, it has been shown that the hippocampus modulates anxiety states [42, 43], in particular it’s anterior part [44, 45]. Moreover, the anterior hippocampal formation predicted affect-focused psychotherapy outcome [43, 46]. Furthermore, it has been shown that there are bidirectional pathways between hippocampus and amygdala [47] and that the ventral hippocampus and the ACC modulate amygdala activation and anxiety behavior [48]. Here we argue that in our study both hippocampus and PAG successfully regulated negative emotional experiences in the OLP group.

Thus, there seem to be remarkable differences when comparing brain networks linked to placebos with and without deception. Placebos with deception seem to modulate our emotional responses for negative pictures by engaging the prefrontal cortex (among other brain regions) [21], which points to the role of expectations as a mechanism. A contribution of the prefrontal cortex has been shown to be crucial for conventional placebo effects [49]. For example, it has been shown that transiently disrupting the DLPFC using transcranial magnetic stimulation blocked placebo analgesia [50]. In contrast, OLP effects seem to rely on structures such as the hippocampi, ACC and PAG rather than frontal brain areas. Thus, other mechanisms than expectation have to explain the way placebos without deception work. This is also supported by the fact that we did not find any correlations of expectation (or belief in OLPs) with self-reported emotional distress or brain activation (but remarkably the placebo-related brain responses were linked to the general belief in the power of placebos, which is in line with previous research [11]).

The lack of a relationship between expectations and self-report or neural measures of emotional distress is also in line with most of the previous studies on OLP effects (e.g., [13]) and might differentiate open- vs. covert placebo effects. For example, it has been shown that higher baseline expectations predicted double-blind placebo responses, while the opposite effect was reported in the open-placebo trial [51]. In accordance with these findings, it has been reported that OLP effects are independent of reported expectations for pain relief [52], suggesting that OLP effects of modulations of pain (or emotional distress) may be based on lower pain control mechanisms, whereas prefrontal brain areas are not engaged. Other studies on OLP effects have also failed to show any positive correlation between baseline expectations and placebo response (e.g., [53, 54]). Thus, different placebo treatments seem to be based on different mechanisms [55]. This is also supported by a recent study that compared OLP with conventional-double-blind placebos and a no-pill control condition in irritable bowel syndrome. The authors found different predictors for the placebo types (with partially opposite effects), suggesting that different psychological mechanisms may be engaged in OLPs and conventionally concealed placebos [56].

Some limitations of our study may apply. First, our sample included only female participants with a limited age, all of them were students. Second, the participants’ rated the emotional distress they felt after viewing the pictures simply by using a 4-button box. Further research should try to include Self-Assessment Manikin (SAM) ratings [26], which would provide much more detailed information. Third, we measured emotional distress by presenting highly negative pictures. The results should be replicated by using other stress inducing paradigms, e.g., the Montreal Imaging Stress Task [57].

Our results contribute to a growing body of research demonstrating beneficial effects of placebos without deception [9], proposing that OLPs may offer a feasible, cost-effective, and ethically justifiable new way to address both clinical and nonclinical symptoms. Since our results do not only rely on self-report measures but provide neural markers of OLP effects, we argue that it is unlikely that the beneficial effects reported in our study are based merely on response bias. Although further research is necessary to address some limitations of our sample (see above), the results suggest that the non-deceptive employment of placebos may be very promising.

## Supplementary information

Supplmental Material

## Data Availability

All data needed to evaluate the conclusions in the paper are present in the paper and/or the Supplementary Materials.
