# Supplementary material for: Neural underpinnings of open-label placebo effects in emotional distress
Source: Neuropsychopharmacology. 2022 Dec 1;48(3):560–6. doi: 10.1038/s41386-022-01501-3 (PMC9852452; doi:10.1038/s41386-022-01501-3)
Supplement: Supplementary file 1 — Supplmental Material [file 41386_2022_1501_MOESM1_ESM.pdf]

Supplementary Material

Neural underpinnings of open-label placebo effects in emotional distress

Michael Schaefer*, Anja Kühnel, Felix Schweitzer, Sören Enge, Matti Gärtner

Medical School Berlin, 12247 Berlin, Germany

S1: Study design

S2: Results of study 1

S3: Results of random effects analysis (study 2) for brain responses when participants viewed negative relative to neutral pictures irrespective of groups

S4: Correlations between brain activation and behavioral measures

S5: Scatterplots of brain responses in PAG with behavioral measures

S6: Presentation for control and OLP group

S7: Questions to examine belief in OLP

S8: Question to examine expectation in nasal spray

S9: Questions to examine general belief in placebos

S10: Instructions of the experimenter (study 2)

S11: IAPS pictures

S1: Study design. Sequence was identical for both experiments.

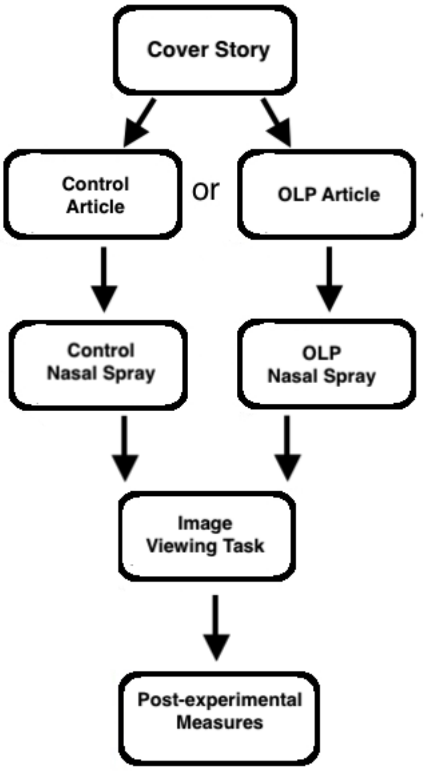

S2: Results of study 1. Bold data points to significant group differences. See text for further details.

	**CTRL group**	**OLP group**	
**N**	54	58	
**females /males**	32/22	33/25	
**age**	23.65 ±7.38	23.41 ±7.32	$t(110) = 0.67$, $p > 0.10$
**evaluation of presentation quality**	5.97 ±1.28	6.25 ±1.13	$t(110) = -1.19$, $p > 0.10$
**expectation (effectiveness of nasal spray)**	0.59 ±1.12	1.23 ±2.02	**$t(110) = -2.05$, $p = 0.021$**
**rating of negative pictures**	5.51 ±1.56	4.85 ±1.49	**$t(110) = 2.30$, $p = 0.011$**
**rating of neutral pictures**	1.32 ±0.48	1.32 ±0.91	$t(110) = -0.03$, $p > 0.10$

S3: Results of random effects analysis for brain responses when participants viewed negative relative to neutral pictures irrespective of groups (whole brain analyses, voxel-wise threshold at $p < 0.001$, uncorrected).

	brain region	peak MNI location (x, y, z)	peak z-value
**Emotional > neutral pictures**	R superior/middle temporal gyrus /angular gyrus	48 -72 0	5.63
	L occipital cortex	-14 -98 0	4.93
	L posterior orbital cortex	-42 22 -18	3.93
	L fusiform gyrus	-42 -36 -18	3.81
	L cerebellum	-20 -80 -22	3.74
	L superior frontal gyrus	-4 56 20	3.74
	R thalamus	18 -30 6	3.71
	R amygdala	26 -6 -22	3.74
	L lingual gyrus	-20 -42 -16	3.37
	R inferior frontal gyrus	52 32 0	3.35
	R caudate	20 -4 22	3.33
**Neutral pictures > emotional pictures**	-	-	-

S4: Correlations between brain activation and behavioral measures (OLP-group, Pearson, two-sided, significant correlations are in bold, statistical trends ($p < 0.10$) in italics).

	Emotional Distress	Expectation	Belief in OLPs	General belief in Placebos	PAG	R Hippo-campus	L Hippo-campus	ACC
**Emotional distress**								
**Expectation in nasal spray**	-0.16							
**Belief in OLPs**	0.20	0.09						
**General belief in Placebos**	-0.19	0.17	0.24					
**PAG**	-0.33	-0.07	0.18	**0.61**				
**R Hippocampus**	-0.22	0.10	0.05	*0.37*	**0.75**			
**L Hippocampus**	-0.21	0.15	0.29	*0.40*	**0.66**	**0.75**		
**ACC**	-0.12	0.05	0.06	-0.09	0.27	**0.47**	**0.51**	

S5: Scatterplots of brain responses in PAG with behavioral measures.

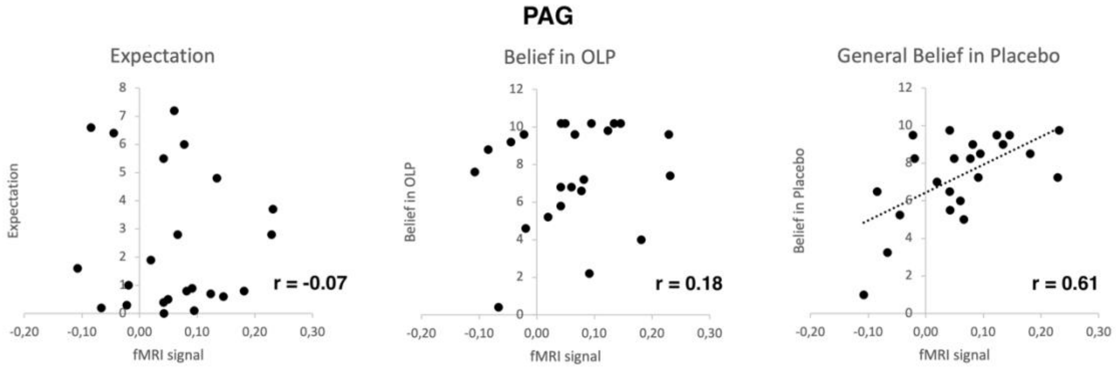

S6: Presentation for OLP and control group

To optimize the placebo manipulation, participants in study 1 and study 2 were asked to read articles on either placebos or pain. The OLP group read an article about placebos, e.g., explaining the participants that placebos are powerful, have been shown to work even without deception, can affect behavior and physiological processes. The control group received a similar article on pain. Both texts were comparable with respect to narrative structure, valanced words, and length. The articles were taken from Guevarra et al. (2020).

The reading manipulation was identical for study 1 and 2, but in study 2 we asked the participants to recall and write about what they remembered from the presentation at the end of the reading (identical to Guevarra et al., 2020).

After the reading manipulation step the nasal sprays were given and the image viewing task started (study 1). In study 2 participants completed the recall task and then also received the nasal spray before entering the fMRI room. The verbal instructions of the experimenter when giving the nasal spray were identical to Guevarra et al. (2020) (for both studies).

S7: Questions to examine belief in OLP

These 5 questions aimed to examine the belief in OLPs and were embedded in other more general belief items on pain. All questions are taken from Guevarra et al., 2020. Participants had to rate the statements on a 11-point scale from 0 (definitely not true) to 10 (definitely true).

"A placebo can still work on me even though I know that I am taking a placebo."
"In order for placebos to work, the person needs to be deceived into believing they are taking an actual medicine."
"A placebo can reduce my negative emotions even though I know I am taking a placebo."
"A placebo only works if the person is deceived into thinking they are taking an actual medicine."
"A placebo can reduce my pain even though I know that I am taking a placebo."

S8: Question to examine expectations with respect to nasal spray

For the control-group the nasal spray was introduced due to technical reasons. Thus, it may be surprising for those participants when asking about relief of emotional distress due to the spray. Therefore, for this group we added the following statement before asking about their expectations: „Sometimes the saline nasal spray has the incidental effect of reducing people's negative emotional response. " Then for both groups we asked to rate how strong they think the nasal spray had reduced their emotional response to the pictures. Subjects had to rate this statement on a visual analogue scale with the ends "not at all" and "very strong".

S9: Questions to examine general belief in placebos

These four items have been taken from Leibowitz et al. (2019) and had to be rated on a 11-point scale from 0 (definitely not true) to 10 (definitely true):

"Placebo effects are a part of all active medications."

“Placebo effects can occur in all illnesses and conditions.”
“Placebo effects happen because the mind has the power to heal.”
“Placebo effects work because placebos influence people's expectations about a particular treatment.”

S10: Instructions of the experimenter (study 2)

Before administering the placebo nasal spray, the experimenter explained to the participants (taken from Guevarra et al., 2020):

Placebo condition:

“From what you have read, you know that placebos are inert substances or procedures that make people feel better mostly because they believe it will. You also know that placebos are powerful and can help reduce pain, depression, anxiety, and negative emotions. On top of that, you’ve read that placebos affect more than how you feel, they actually can change your behavior, physiology, and even brain activity. And new research has also shown that placebos can work even if you know you are taking one since the key ingredient is the positive belief that it can help and that it works.“ Then the administration provides the nasal spray and explains further: “I just want to remind you that I just administered a placebo nasal spray that contains no active ingredients, but if you believe it will reduce your negative emotional reactions to these images, then it will. The placebo really works because of your positive beliefs and expectations.”

Control condition:

“Your sinuses consist of passageways that surround the nasal cavity. Two of these passageways are located above and under your eyes. One is located between the eyes. The last one is located behind them. The nasal cavity is a large space above and behind the nose in the middle of the face. These spaces are naturally filled with a thick membrane that gets recycled throughout the day. Unfortunately, this thick membrane often interferes with the metabolic signals produced by your brain. So, to help us get better signals, we administer a saline nasal spray to help clear some of this space. “

S11: IAPS images

Picture Type	Number	Picture Type	Number	Picture Type	Number
Neutral	2190	Neutral	2200	Neutral	2210
Neutral	2230	Neutral	2570	Neutral	2840
Neutral	5500	Neutral	5531	Neutral	7000
Neutral	7002	Neutral	7009	Neutral	7010
Neutral	7020	Neutral	7025	Neutral	7035
Neutral	7050	Neutral	7080	Neutral	7100
Neutral	7150	Neutral	7160	Neutral	7170
Neutral	7175	Neutral	7190	Neutral	7217
Neutral	7224	Neutral	7233	Neutral	7235
Neutral	7550	Neutral	7700	Neutral	5950
Neutral	6150	Neutral	7006	Neutral	7009
Neutral	7035	Neutral	7080	Neutral	7100
Neutral	7150	Neutral	7190	Neutral	7233
Neutral	7235	Neutral	7250	Neutral	7502
Neutral	7545	Neutral	7550	Neutral	7700
Negative	2688	Negative	6312	Negative	6313
Negative	6825	Negative	9425	Negative	9428
Negative	9620	Negative	9622	Negative	9908
Negative	3181	Negative	3350	Negative	3500
Negative	3530	Negative	6212	Negative	6821
Negative	2683	Negative	2811	Negative	3301
Negative	6550	Negative	6520	Negative	8485
Negative	9050	Negative	9183	Negative	9414
Negative	6242	Negative	6231	Negative	6230
Negative	3170	Negative	3220	Negative	9903
Negative	3130	Negative	3140	Negative	3150
Negative	3170	Negative	3180	Negative	3350
Negative	3400	Negative	3550	Negative	6230
Negative	6350	Negative	6360	Negative	6510
Negative	6550	Negative	6560	Negative	6570
